# Indole–Imidazole Hybrids as Emerging Therapeutic Scaffolds: Synthetic Advances and Biomedical Applications

**DOI:** 10.3390/molecules30214164

**Published:** 2025-10-23

**Authors:** Wafa A. Bawazir, Qurratul Ain

**Affiliations:** Department of Chemistry, Faculty of Science, King Abdulaziz University, Jeddah 21589, Saudi Arabia; wbawazeer@kau.edu.sa

**Keywords:** indole–imidazole scaffolds, heterocyclic compounds, synthetic strategies, therapeutic applications, medicinal chemistry

## Abstract

Indole and imidazole structures are widely used in medicinal chemistry for their unique electronic, steric, and pharmacophoric qualities that drive diverse biological effects. Combining indole and imidazole structures enhanced structural diversity, binding affinity, making a promising approach for creating multifunctional therapeutic agents. This review presents a comprehensive overview of the synthetic strategies developed for indole–imidazole derivatives, encompassing multistep synthesis, one-pot multicomponent condensation reactions, metal-catalyzed reactions, metal-free catalysis, and various green chemistry approaches, with particular emphasis on efficiency, yields, and practical limitations. In addition, this review critically evaluates the biological activities of indole–imidazole scaffolds, highlighting their applications as anticancer, antioxidant, anti-microbial, neurological, and metabolic agents. By integrating recent synthetic advances with pharmacological insights, this review underscores both the opportunities and challenges in the hybrid design. It also provides direction for future research aimed at developing novel drug candidates to tackle current healthcare concerns such as antibiotic resistance, cancer, and chronic diseases.

## 1. Introduction

The importance of nitrogen-containing heterocyclic compounds in medicinal chemistry is well established, as they form the structural backbone of many marketed drugs and bioactive molecules [1]. Among these, indole and imidazole scaffolds occupy a privileged position due to their diverse pharmacological properties and ability to interact with a wide range of biological targets. Indole derivatives are found in natural products and therapeutic agents with activities ranging from anticancer and anti-microbial to anti-inflammatory and antidepressant effects [2,3,4,5]. They are also used to manage infectious diseases, metabolic disorders, and neurodegenerative disorders [6]. Indoles with well-defined functional groups can be prepared using innovative photochemical and electrochemical techniques with nitroarenes as the starting material. Beyond their inherent biological activity, indoles function as versatile intermediates for derivatization into scaffolds such as isatins and oxindoles, thereby broadening their synthetic utility [7,8]. Several indole-based compounds, such as indomethacin, pindolol, and indapamide, have already been approved as drugs [9]. Likewise, imidazole derivatives exhibit broad utility, attributed to their amphoteric nature, hydrogen-bonding capacity, and the ability to coordinate with biomolecules, making them indispensable in anti-fungal, antibacterial, anticancer, antihypertensive, antioxidant [10], antiulcer, analgesic, anti-inflammatory [11], and cardiovascular drugs, various agrochemicals, artificial receptors, and biomimetic catalysts [12,13,14,15]. Imidazole derivatives can be prepared by simple one-pot condensation techniques [10,11,16]. USFDA-approved imidazole-based drugs include ketoconazole, clotrimazole, miconazole, and metronidazole [17].

**Figure 1:**
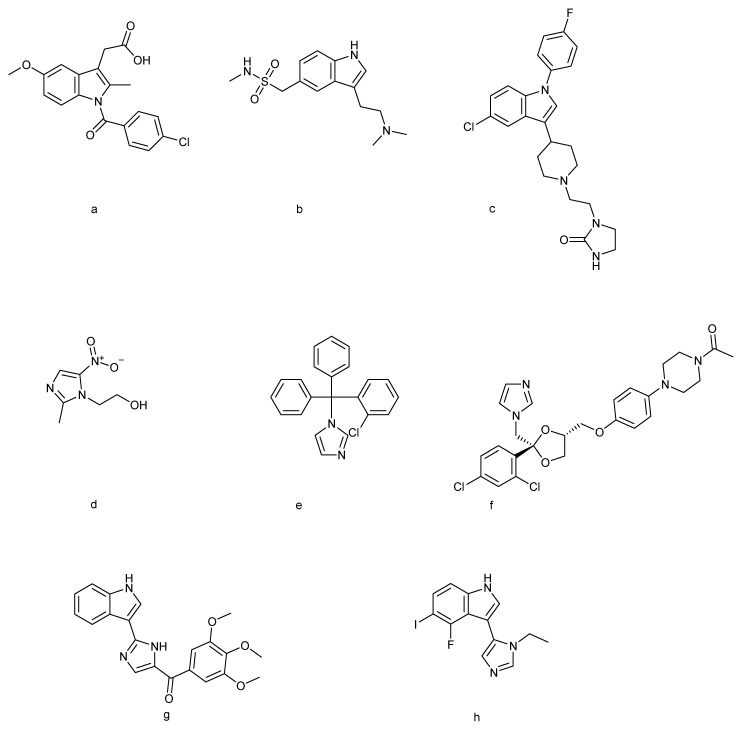
Indole-based drugs [35]: (**a**) indomethacin, (**b**) sumatriptan, and (**c**) sertindole; imidazole-based drugs [17]: (**d**) metronidazole, (**e**) clotrimazole, and (**f**) ketoconazole; indole–imidazole-based drugs [36,37]: (**g**) sabizabulin and (**h**) AGH-192.

In recent years, the indole–imidazole scaffolds, which merge the structural advantages of both heterocycles, have gained significant attention in drug discovery and chemical biology. The hybridization of indole and imidazole rings enhances the binding affinity [18], modulates the physicochemical properties, and allows structural diversity through substitution at multiple positions. This structural versatility has enabled the development of molecules with potent activities against cancer [19], microbial infections, neurological disorders [20], and metabolic diseases. Moreover, several indole–imidazole derivatives have progressed into pre-clinical and clinical evaluation, underscoring their translational potential. Figure 1 displays various FDA-approved drugs containing indole and imidazole moieties used for diverse therapeutic applications, along with indole–imidazole analogues currently undergoing pre-clinical and clinical trials.

On the synthetic front, advances in methodologies, such as multicomponent reactions [20,21,22], transition-metal-catalyzed couplings [19,22,23], and green chemistry approaches, have facilitated efficient access to indole–imidazole frameworks [24,25,26]. These strategies not only expand the chemical space but also overcome the traditional challenges of regioselectivity [27,28], low yields, and purification difficulties. Despite these advances, limitations remain, including poor scalability and limited structure–activity relationship (SAR) data for certain analogues. Addressing these challenges will provide opportunities for further optimization and innovation.

Several reviews have summarized the importance of indole- and imidazole-based compounds in medicinal chemistry. For instance, the authors of [2,7,9,29,30] focused primarily on indole derivatives and their pharmacological activities, while the authors of [13,14,15,21,31] emphasized imidazole-containing drugs and their therapeutic relevance. More recent reviews, such as the study in [1], have discussed hybrid heterocycles but provided limited coverage of indole–imidazole conjugates; the authors of [19] described derivatives often involving substitutions at a few positions (e.g., indole C-3), while other regions of the scaffold remained unexplored. The authors of [32] concentrated on a single therapeutic area: anticancer activity. In [33], only articles from Jan 2015 to May 2020 were reviewed; more recent studies (post-2020) were missing. Finally, in [34], biological applications, therapeutic potential, and SAR were not discussed; the review was purely synthetic. To the best of our knowledge, no comprehensive review exists that systematically integrates the synthetic strategies, structural modifications, therapeutic diversity, and challenges associated with indole–imidazole derivatives.

The novelty of this review lies in the following: (i) providing an updated and consolidated overview of synthetic methodologies (including multicomponent reactions, metal-catalyzed couplings, and green protocols); (ii) highlighting the therapeutic spectrum of indole–imidazole hybrids across cancer, microbial, neurological, and metabolic diseases; (iii) critically assessing the advantages and limitations of existing strategies; and (iv) outlining future prospects for the rational design of these scaffolds. By addressing the chemical and biological aspects in an integrated manner, this review aims to serve as a timely resource for medicinal chemists and drug discovery researchers.

## 2. Synthetic Approaches for Indole–Imidazole Derivatives

### 2.1. Multistep Synthetic Methods

#### Orthogonal Protection for Multistep Synthesis

**Scheme 1:**
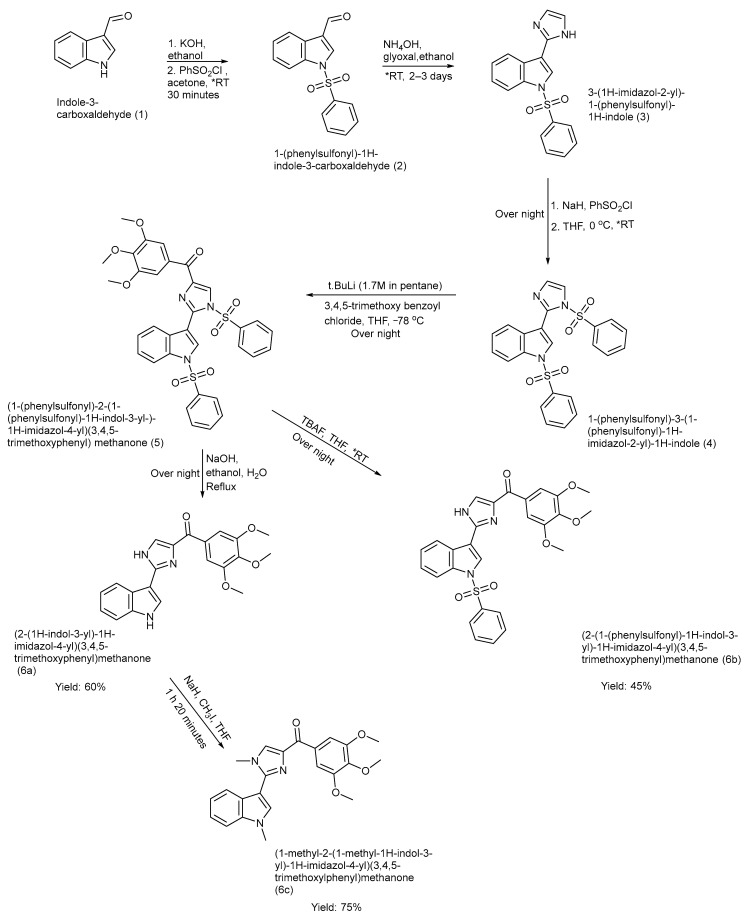
Synthesis of ABI-III analogues using orthogonal protection multi-step method. (* RT—room temperature).

Chen et al. designed and synthesized three indole–imidazole derivatives **6** (**a–c**) using the ring construction method [38]. These ABI-III (2-aryl-4-benzoyl-imidazol) compounds were synthesized after focused structure–activity relationship studies of previously discovered ABI-I and ABI-II analogues. Three novel compounds were synthesized **6** (**a–c**) with improved efficacy, with hydrogen, a methyl group, and a bulkier phenyl sulphonyl group attached to the indole nitrogen atom, respectively [38]. The yields were product (**6a**): 60%, product (**6b**): 45%, and product (**6c**): 75%. The synthetic pathway, reagents, reaction time, and conditions are illustrated in Scheme 1 [38].

The study presented several strengths, including the development of potent tubulin polymerization inhibitors with nanomolar activity and validated via cell-based assays. Significantly, these compounds are not substrates of P-glycoprotein (P-gp), thus overcoming multidrug resistance. The work was supported by SAR, molecular docking studies, and thorough chemical characterization. However, there were also some limitations: it lacked in vivo pharmacokinetic profile, toxicity, and efficacy data, and provided a limited assessment of the selectivity toward normal cells [38].

Applications:

The above synthesized analogues (**6a–6b**) were tested against a panel of human metastatic melanoma cell lines (A375 and WM164) and human prostate cancer lines (LNCaP, PC-3, and Du 145). They showed significant anticancer activity with a half-maximal inhibitory concentration (IC_50_) in nM, as given in Table 1 below, via inhibition of tubulin polymerization. In addition, they were not a substrate for Pgp, and they were able to effectively overcome P-gp-mediated multidrug resistance. This hypothesis was supported by using the paclitaxel-resistant cell line PC-3/TxR. Table 2 shows the IC_50_ of ABI-III analogues on paclitaxel-resistant cell lines [38].

The results showed that (**6a**) proves to be a highly potent anticancer agent among three analogues, while (**6b**) had a bulkier phenyl sulphonyl group attached to the indole ring, which resulted in 50 times lower anticancer activity. Likewise, substitution of indole-NH or imidazole-NH with a smaller functional group resulted in well-tolerated, significant anticancer activity, as shown by (**6c**). The ability of (**6a–6c**) to inhibit tubulin polymerization while evading P-gp-mediated drug efflux distinguishes them from classical anticancer drugs like paclitaxel, which shows a high resistance index due to efflux by P-gp [38].

### 2.2. One-Pot Multicomponent Reaction Methods

#### 2.2.1. Ring Construction Methods

**Scheme 2:**
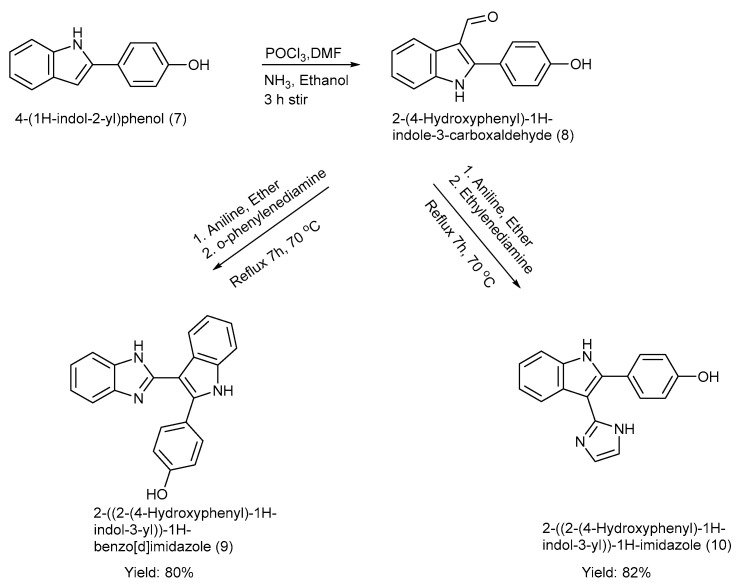
Synthesis of 2-phenyl indole derivatives using ring construction method.

El-Nakkady et al. [39] synthesized two indole–imidazole derivatives (**9–10**) using ring construction methods incorporating the Vilsmeier–Haack reaction with yields of product (**9**) at 80% and product (**10**) at 82% [39]. The 2-phenyl indole moiety was formylated at C-3, and the formyl group was replaced with several other substituents, including imidazole and benzimidazole, respectively [39]. The synthesis process, along with the reagents and reaction conditions, is depicted in Scheme 2 [39].

The strength of this work is the potent anti-tumour activity of the synthesized scaffolds, rational design, and well-described synthetic routes. Nevertheless, the work was restricted to in vitro assays without in vivo assessment, pharmacokinetic, or toxicity profile, leaving the clinical relevance uncertain. Moreover, the selectivity, resistance mechanisms, solubility, and synthetic scalability were also insufficiently addressed [39].

Applications:

The target substances were tested against breast cancer cell lines, MCF-7 and MDA-MB-231, via the colchicine binding site of tubulin, as depicted in Table 3. The results showed better anti-tubulin activity against MCF-7 cancer cells as compared to the drug Vincristine; however, when the same compounds were further tested against MDA-MB-231, they did not show significant anti-tubulin activity. The results depicted that the moiety with benzimidazole (**9**) showed better anti-tubulin activity than the one with imidazole (**10**). Hence, product (**10**) can be considered as a potential anticancer agent for future endeavours [39].

**Scheme 3:**
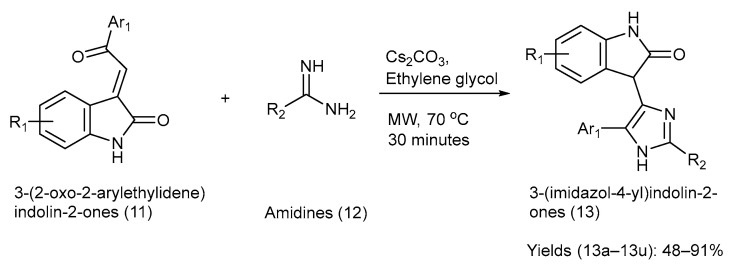
Synthesis of 3-(Imidazole-4-yl) Indolin-2-ones using [3+2] cyclization method.

Chen et al. [27] developed a [3+2] cyclization process to regioselectively synthesize 3-(Imidazole-4-yl) Indolin-2-ones. The starting materials used for the reaction were 3-(2-Oxo-2-ethylidene) indolin-2-ones [27]. The chemical reaction, reagents, reaction time, and conditions involved in the synthesis are shown in Scheme 3. Various substituents on the starting materials (**11**) and (**12**), along with the synthesized indole–imidazole analogues (**13**), and their respective yields are presented in Table 4 [27].

The starting material (**11**) had multisite reactivity and amidines (12), were also variously substituted. Thus, it resulted in polyfunctionalized 3-(Imidazole-4-yl) Indolin-2-ones, making it attractive for generating compound libraries in drug discovery. The method used mild conditions, with Cs_2_CO_3_ in ethylene glycol under microwave irradiation, offering notable speed and simplicity. However, the study did not report any biological evaluation, leaving the pharmacological relevance of the products unknown. Furthermore, the scalability in terms of the microwave conditions and solvent choice may restrict direct industrial applications [27].

Application:

Several studies have highlighted substituted indolin-2-ones as ligands for the dopamine D4 receptor [40] and α-synuclein fibrils [41], while others have reported their antibacterial [42], anticonvulsant [43], and Aurora B kinase [44] or bromodomain inhibitory activities [45].

#### 2.2.2. Metal-Catalyzed Coupling Methods

**Scheme 4:**
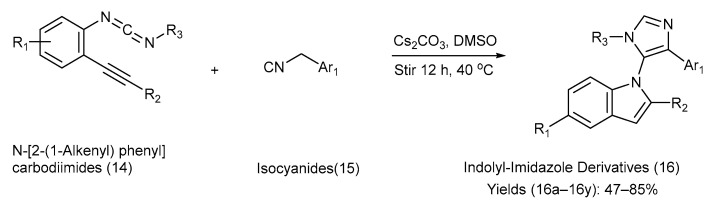
Synthesis of indolyl-imidazole derivatives using metal-catalyzed coupling method.

Hao et al. [46] reported a versatile approach for the synthesis of indole–imidazole analogues via a base-promoted tandem reaction between N-[2-(1-alkynyl) phenyl] carbodiimides and isocyanides, carried out in the presence of cesium carbonate and dimethyl sulfoxide (DMSO) at 40 °C. The reactions included the [3+2] cyclization reaction between isocyanides and N-[2-(1-alkynyl) phenyl] carbodiimides. Carbodiimides feature dual sites of reactivity, with the central carbon atom acting as an electrophile and the terminal nitrogen atom serving as an electron-rich centre. Owing to recent progress in carbodiimide chemistry, N-[2-(1-alkynyl) phenyl] carbodiimides are expected to play a pivotal role as intermediates in the construction of structurally privileged N-heterocycles. Hao and co-workers synthesized 25 different indole–imidazole derivatives in moderate-to-good yields using carbodiimides, thus diversifying natural-product-based libraries [46]. Scheme 4 presents the chemical reaction, reagents, and reaction conditions involved in the synthesis. Table 5 lists the different substituents present on the starting materials (**14**) and (**15**), along with those on the indole–imidazole product (**16**) and the yield of each product [46].

The advantages of the reported synthetic method include a concise, economical one-pot tandem protocol, relatively mild reaction conditions, and the ability to build structural complexity in a single operation. The method used a range of substrates, thus offering flexibility in substituent variation. However, biological evaluation of the products was lacking; so, their utility in medicinal chemistry remains untested. In addition, the yields were only moderate in some cases, and the method may face limitations in purification, depending on the substituents [46].

**Scheme 5:**
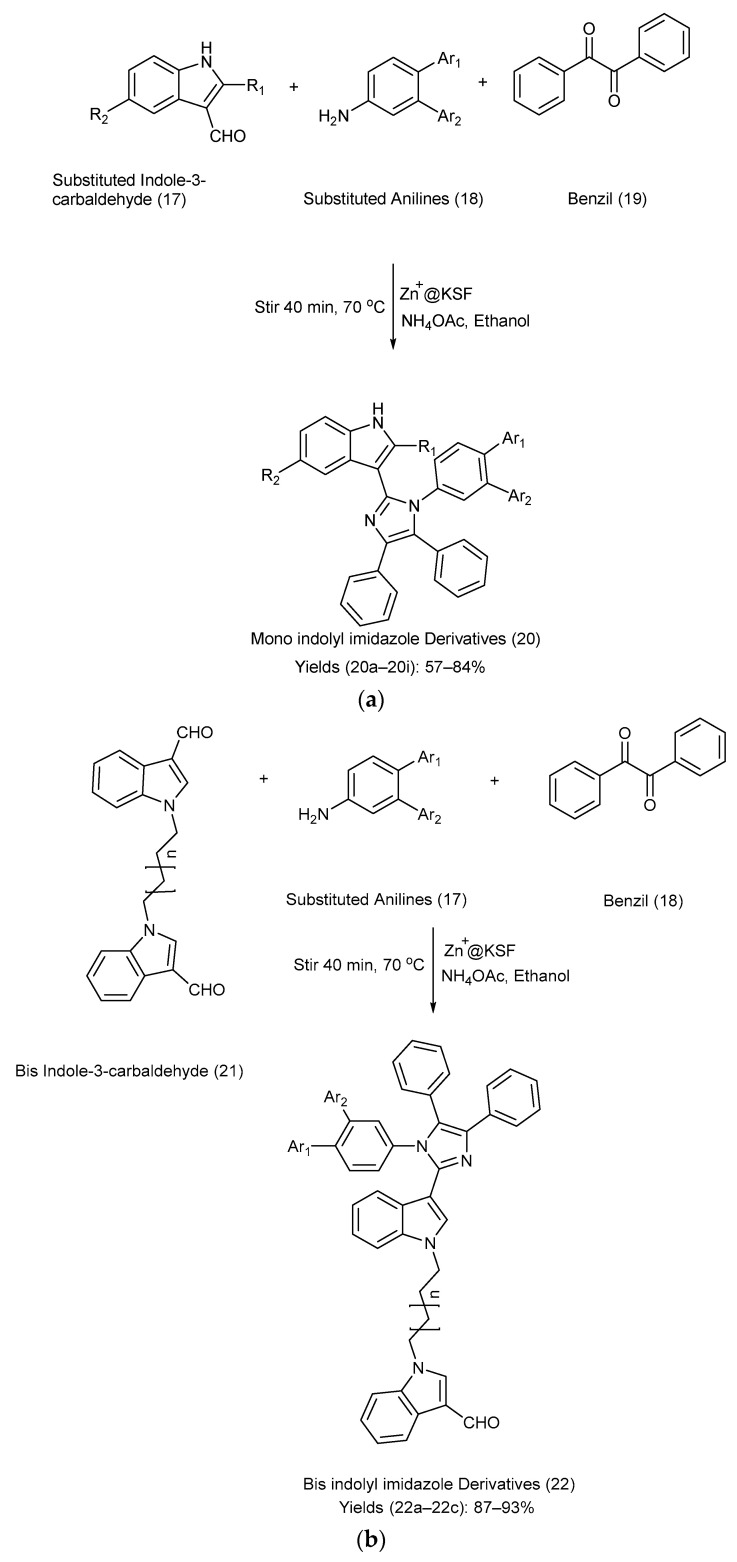
(**a**). Synthesis of mono-indolyl-imidazole derivatives using metal-catalyzed coupling method. (**b**). Synthesis of bis-indolyl-imidazole derivatives using metal-catalyzed coupling method.

Mahmoodi et al. [22] reported a powerful, efficient, reliable, and highly selective method for the synthesis of mono-indolyl imidazole and bis-indolyl imidazole frameworks using Zn^+2^ supported on montmorillonite KSF. Indole-3-carbaldehydes were allowed to react with substituted anilines, benzil, using Zn^+2^ on KSF, ammonium acetate, and ethanol via nucleophilic addition and condensation reactions [22]. Refer to Scheme 5a,b for the reagents used, reaction setup, and detailed synthetic methods of the indolyl imidazole derivatives, respectively. Table 6 illustrates the substitution patterns for the starting compounds (**17**), (**18**), and (**21**) and their corresponding indole–imidazole derivatives (**20**) and (**22**), as well as their respective yields [22].

The method is efficient and environmentally friendly, and the catalyst is recyclable, aligning with green chemistry principles. Column chromatography was not required for this method. The antibacterial evaluation revealed that some derivatives exhibit promising activity against the selected bacterial strains. However, the study lacked detailed mechanistic insights and did not fully explore the antibacterial spectrum or potential cytotoxicity. Moreover, the scalability and the practical application of the catalyst in industrial settings were unaddressed [22].

Applications:

The above synthesized bis- and mono-indolyl imidazoles were assessed for antibacterial activity against various Gram-positive and Gram-negative bacteria, including *Pseudomonas aeruginosa* (*P. aeruginosa*), *Salmonella enteritis* (*S. enteritis*), *Bacillus subtilis* (*B. subtilis*), and *Micrococcus luteus* (*M. luteus*). A total of 100 µg in 0.1 mL DMSO solution of each compound was prepared, and zones of inhibition for each bacterium were checked and compared to commonly used antibiotics, i.e., erythromycin and tetracyclin. The results are mentioned in Table 7. All the compounds exhibited significant antibacterial activity. However, the mono-indolyl imidazoles showed higher anti-microbial activity than the bis-indolyl imidazoles [22].

#### 2.2.3. Metal-Free Coupling Methods

**Scheme 6:**
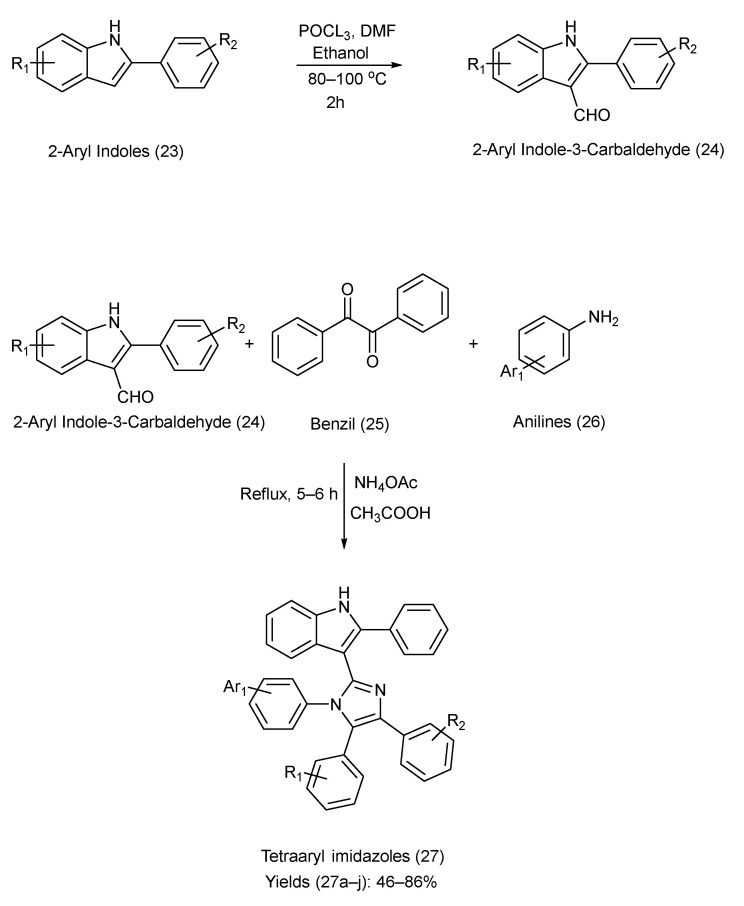
Synthesis of tetra aryl imidazole–indole Derivatives using Metal-Free Coupling Method.

Naureen et al. [47] prepared tetra aryl imidazole and indole derivatives using multiple components. The reactants used were 2-arylindole-3-carbaldehydes (synthesized using the Vilsmeier–Haack reaction [48,49], via formylation of 2-aryl indoles (synthesized via Fischer indole synthesis [50])), ammonium acetate, benzil, and substituted anilines. Benzil and anilines were used to construct the imidazole ring on the 3-position of 2-arylindole-2-carbaldehydes in the presence of NH_4_OAc and CH_3_COOH [47]. Scheme 6 outlines the reagents, reaction time, reaction conditions, and chemical reactions used in the synthesis. A list of the substituents on precursors (**23**) and (**24**), the product (**27**), and the yield of each product is provided in Table 8 [47].

The reaction procedure was simple, incorporating one-pot synthesis with easily available solvents, enabling rapid access to structurally diverse tetra aryl imidazoles. Biological evaluation revealed that the scaffolds possess potent anti-urease activity and notable antioxidant potential, underscoring their pharmacological relevance. However, the study provided limited mechanistic insight and lacked data about in vivo testing, as well as broader assessments of toxicity and selectivity [47].

Applications:

The newly synthesized compounds showed significant anti-urease activity but were not proven to be good radical scavengers/antioxidants. Table 9 shows the anti-urease and antioxidant activity of tetra aryl imidazole–indole derivatives, indicating the percentage inhibition at a concentration of 0.5 mM and IC_50_ (µM) of each analogue synthesized. The decreasing order of anti-urease activity was **27j** > **27g** > **27h** > **27i** > **27f** > **27d** > **27c** > **27e** > **27a** > **27b**, whereas only **27a–27f** were active radical scavengers, with decreasing order of antioxidant activity being **27b** > **27d** > **27f** > **27a** > **27c**. In general, the results indicated that compounds with electron-donating functional groups, such as halogens and methyl groups, act as more potent agents for anti-urease activity. The data also suggested that the compounds are weak antioxidants as compared to Quercetin [47].

**Scheme 7:**
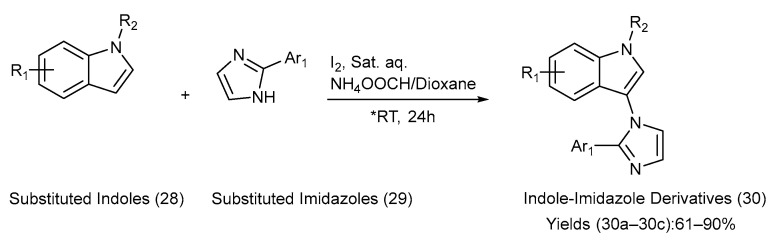
Synthesis of C-N-coupled indole–imidazole derivatives using metal-free coupling method. (* RT—room temperature).

Wu et al. [51] developed an efficient, regioselective, and metal-free coupling reaction for the synthesis of three indole–imidazole derivatives. The substituted indoles and imidazoles underwent C-N coupling using iodine and a saturated aqueous solution of NH_4_OOCH in 1,4-dioxane at room temperature [51]. This method was effective for both free and alkyl-substituted indoles, leading to the synthesis of a diverse series of novel indole–imidazole derivatives. The yields were moderate-to-excellent, and the pH significantly affected the yield. Hence, the pH environment was closely monitored. Scheme 7 presents the chemical transformations employed in the synthesis. Table 10 lists the substituents on the starting materials (**28**) and (**29**), the indole–imidazole derivatives formed in reaction (**30**), and the yields of the derivatives [51].

This synthetic approach is environmentally friendly, avoids toxic metal catalysts, and proceeds under mild conditions, making it practical and sustainable. It demonstrates regioselectivity, and the broad substrate scope allows efficient access to diverse indole derivatives. However, the reported derivatives were not biologically evaluated; so, the pharmacological potential of the products remains untested [51].

**Scheme 8:**
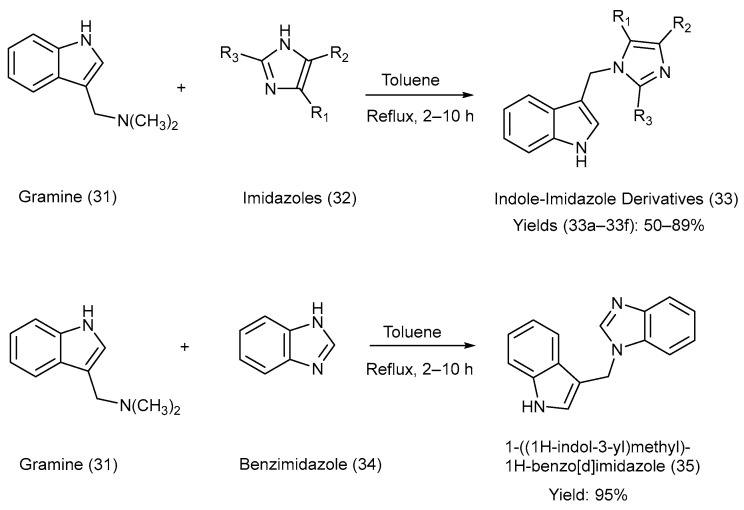
Synthesis of C-3-substituted indolyl methane–imidazole derivatives using metal-free coupling method.

Jasiewicz et al. [19] synthesized C-3-substituted indole derivatives from gramine [3-(dimethyl amino methyl) indole], an alkaloid present in barley (*Hordeum vulgare*), via a nucleophilic substitution reaction. Gramine with a good leaving group was used as a substrate, and various alkyl-substituted imidazole moieties were heated at reflux in the presence of toluene [19]. The reactions utilized for the synthesis, reaction reagents, and reaction parameters are illustrated in Scheme 8. Various substituents attached to precursors (**32**) and the products (**33**), as well as the yields of products (**33**) and (**35**), are presented in Table 11 [19].

The method was a simple, innovative hybridization strategy, using starting materials from natural origins, and it did not require column chromatography. The synthesized derivatives were comprehensively characterized (spectroscopy and crystallography) and biologically evaluated, covering antioxidant, cytoprotective, antibacterial, and fungicidal effects. However, the work was limited to a relatively small library of only ten compounds, and there was a lack of in-depth mechanistic or molecular docking studies and an absence of in vivo validation to establish therapeutic relevance. Moreover, the cytotoxicity against normal mammalian cells was not fully explored, preventing conclusions on safety for drug development [19].

Applications:

The synthesized indolyl methane–imidazole derivatives had a wide range of therapeutic effects. They showed excellent antioxidant properties when the chelating activity was assessed based on the inhibition of Fe^2+^–ferrozine complex formation, following incubation of the synthesized scaffolds with Fe^2+^. Compounds **33** (**a–d**) proved to be the best chelators, compound (**33e**) did not show any activity, (**33f**) had moderate chelating activity, and (**35**) also showed negligible chelating activity. They also proved to be good cytoprotective agents when evaluated against oxidative hemolysis induced by free radicals generated by AAPH (2,2′-azobis(2-amidinopropane) dihydrochloride). Compounds **33** (**a–d**) with electron-donating substituents showed the least hemolytic activity; hence, they can be considered for further evaluation as they proved to be biocompatible compounds. While compounds **33** (**e–f**) showed slightly higher hemolytic activity, (**35**) was the highest with 23% hemolytic activity and was not a biocompatible compound. The cytoprotective property was based on electron-donating or electron-withdrawing groups attached to the indole and imidazole rings. Compounds **33** (**a–d**) have electron-donating substituents, and the cytoprotective activity is in the order **33a** > **33b** > **33c** > **33d**. (**33f**) showed a bit higher cytoprotective activity, while (**33e**) and (**35**) showed the least cytoprotective activity [19].

Indolyl methane with imidazoles also showed significant antibacterial activity when tested against *Bacillus subtilis* (*B. subtilis*), *Micrococcus luteus* (*M. luteus*), *Escherichia coli* (*E. coli*), and *Pseudomonas fluorescens* (*P. fluorescens*). Table 12 displays the antibacterial activity of the analogues by determining the zones of inhibition in mm of each bacterial strain. The data showed that compounds (**33b**) and (**33f**) exhibit strong antibacterial effects, while the rest of the compounds did not show significant antibacterial activity. The synthesized agents were also studied for their anti-fungal activity against four strains, i.e., *Coiolus versicolor* (*C. versicolor*), *Poria placenta* (*P. placenta*), *Coniophora puteana* (*C. puteana*), and *Gloeophyllum trabeum* (*G. trabeum*). All the compounds showed 100% inhibition at 0.1% concentration. Among all the tested products, (**33e**) showed significant anti-fungal activity against *P. placenta* and *C. puteana,* which are resistant to commonly used fungicides. Table 13 lists the percentage concentration of each compound used and percentage inhibition of each fungal strain, respectively [19].

Another notable feature was the good ADME (absorption, distribution, metabolism, and excretion) parameters of these derivatives; therefore, they can be considered good agents for the development of novel antioxidants [19].

#### 2.2.4. Green Chemistry Approaches

**Scheme 9:**
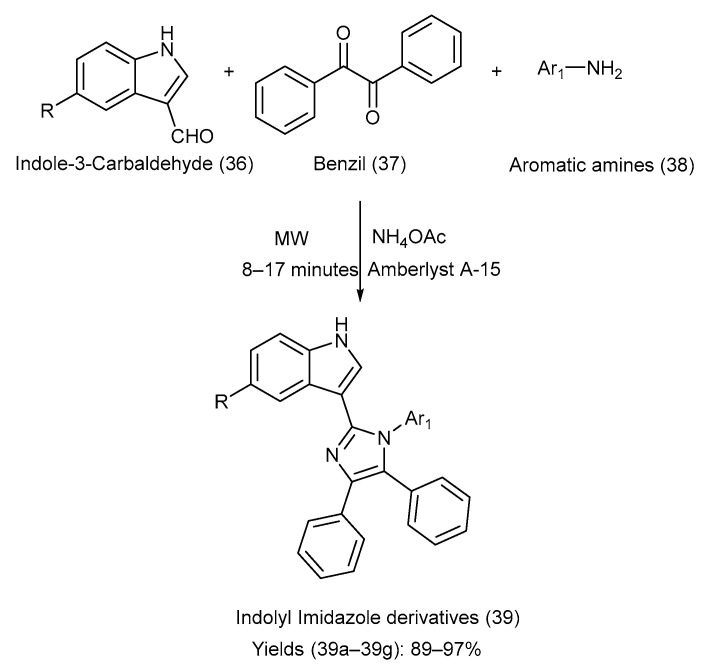
Synthesis of indolyl-imidazole derivatives via condensation under solvent-free conditions using Amberlyst A-15 catalyst.

Nirwan et al. [52] synthesized eight different indole–imidazole derivatives via condensation of indole-3-carbaldehyde, various substituted aromatic amines, and ammonium acetate in the presence of the Amberlyst A-15 catalyst via irradiation with microwaves under solvent-free conditions [24,25,52]. The synthetic pathway with reaction conditions is depicted in Scheme 9. Table 14 presents the different groups attached to the starting materials (**36**) and (**38**), the indole–imidazole derivative (**39**), and the comparison of their yields [24,25,52].

The mentioned one-pot multicomponent methodology was eco-friendly and cost-effective, with short reaction times, excellent yields, and a reusable catalyst, thus making the strategy sustainable and practical. The synthesized derivatives were well characterized (FTIR, NMR, and MS) and showed promising antibacterial and anti-fungal activities. The study had certain limitations, including the relatively small number of synthesized compounds, the lack of molecular-level biological evaluation, and the lack of an in vivo evaluation to confirm the pharmacological relevance [24,25,52,53].

Applications:

The indolyl imidazole derivatives synthesized were tested against four bacterial strains, i.e., *Staphylococcus aureus* (*S. aureus*), *Staphylococcus epidermidis* (*S. epidermidis*), *Escherichia coli* (*E. coli*), *Pseudomonas aeruginosa* (*P. aeruginosa*), and one fungal strain, i.e., *Candida albicans* (*C. albicans*), as depicted in Table 15. Among the tested compounds, (**39c**) showed significant antibacterial activity, showing maximum zone of inhibition for *S. aureus* when compared to Ampicillin and Amikacin. However, the products did not exhibit significant anti-fungal activity as compared to fluconazole [53].

**Scheme 10:**
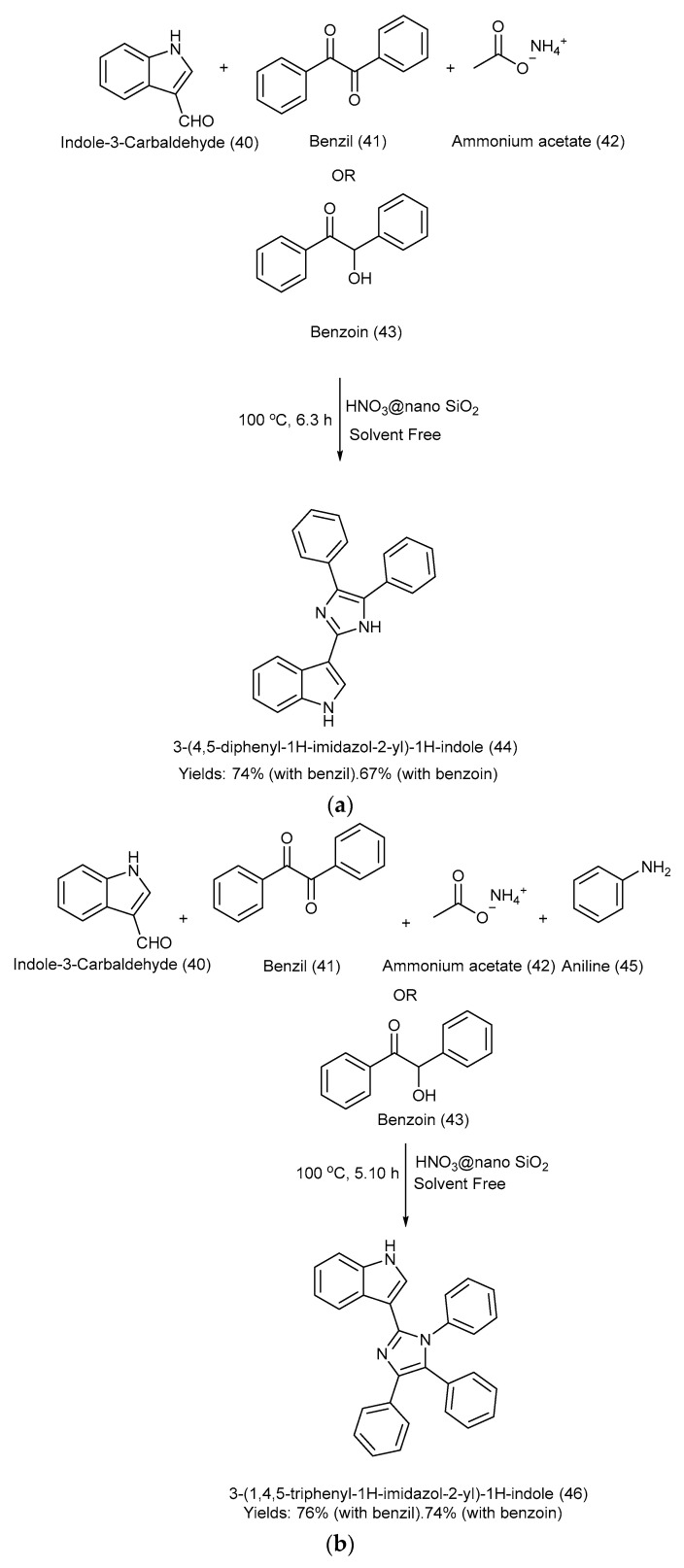
(**a**). Synthesis of 3-(4,5-diphenyl-1H-imidazol-2-yl)-1H-indole via condensation under solvent-free conditions using HNO_3_@nano SiO_2_ catalyst. (**b**). Synthesis of 3-(1,4,5-triphenyl-1H-imidazol-2-yl)-1H-indole via condensation under solvent-free conditions using HNO_3_@nano SiO_2_ catalyst.

Nikoofar et al. [26] developed a simple and efficient one-pot multicomponent methodology to synthesize 3-(4,5-diphenyl-1H-imidazol-2-yl)-1H-indole and 3-(1,4,5-triphenyl-1H-imidazol-2-yl)-1H-indole via cyclization and the condensation of benzil/benzoin, 1H-indole-3-carbaldehyde, ammonium acetate, and amine using a heterogeneous and eco-friendly catalyst, nitric acid, supported on nano silica at 100 °C under solvent-free conditions. The yields were as follows: product (**44**) with benzil: 74%, and with benzoin-67%; product (**46**) with benzil: 76%, and with benzoin: 74% [26]. Scheme 10a,b outlines the methods used for the formation of 3-(4,5-diphenyl-1H-imidazol-2-yl)-1H-indole and 3-(1,4,5-triphenyl-1H-imidazol-2-yl)-1H-indole, respectively, along with the reagents used and reaction conditions [26].

The methodology incorporated nano acid catalysis that led to high atom efficiency, a convenient technique for product purification, high yields, short reaction times, an eco-friendly solvent-free medium, and regeneration of the catalyst. However, the study was limited to a small number of tested recycling cycles (only three) for the catalyst, the absence of industrial applicability studies, and a lack of evaluation of the synthesized derivatives [26].

Applications:

The scaffolds synthesized above can be evaluated for antibacterial, anti-fungal, antioxidant, and various other biological applications [21,47,52].

**Scheme 11:**
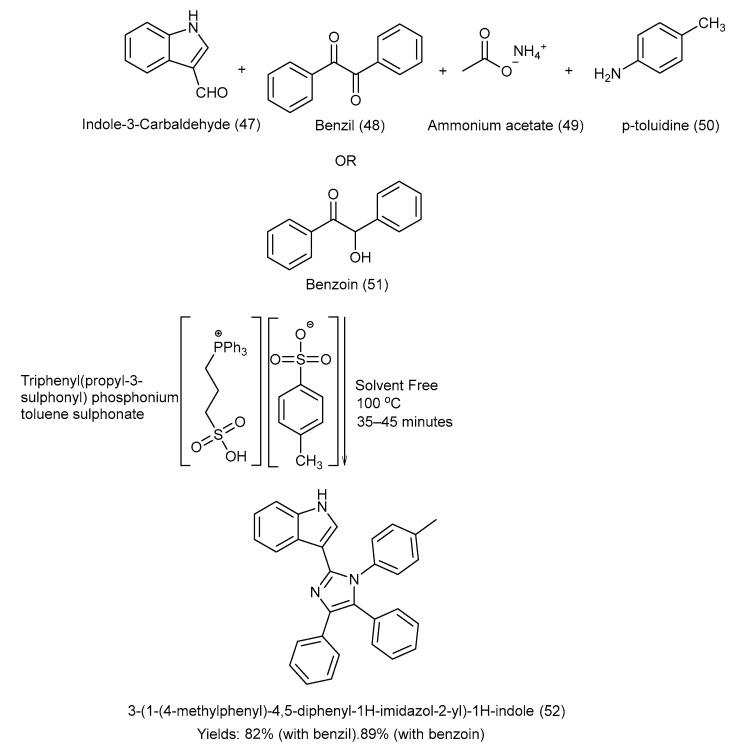
Synthesis of 3-[1-(4-methylphenyl)-4,5-diphenyl 1H-imidazol-2-yl]-1H-indole via condensation under solvent-free conditions using TPSPT catalyst.

Shaterian et al. [54] synthesized a Bronsted acid-liquid reusable catalyst, i.e., triphenyl(propyl-3-sulphonyl) phosphonium toluene sulfonate (TPSPT), and used it to synthesize 3-[1-(4-methylphenyl)-4,5-diphenyl 1H-imidazol-2-yl]-1H-indole via the coupling reaction of benzil/benzoin with indole-3-carbaldehyde and p-toluidine in a solvent-free environment at 100 °C. Ionic liquid technology is an innovative and environmentally safe approach for the synthesis of indole–imidazole derivatives. This one-pot four-component synthetic strategy was quite efficient and provided good yields, i.e., product (**52**) (with benzil: 82%, with benzoin: 89%) [54]. The complete synthetic approach, chemical reagents, and experimental conditions are shown in Scheme 11 [54].

This method demonstrated high efficiency, mild reaction conditions, short reaction times, excellent yields, and the reusability of the ionic liquid catalyst, which makes the method cost-effective and environmentally friendly. Ionic liquid technology is an innovative and environmentally safe approach for the synthesis of indole–imidazole derivatives. It also avoids the need for hazardous mineral acids, aligning with green chemistry principles. Nonetheless, the study was subject to certain limitations, including the lack of detailed studies on the catalyst stability over many recycling runs, the high cost of ionic liquid preparation compared to conventional catalysts, and limited biological evaluation data of the synthesized imidazoles, restricting its broader application in therapeutics or industrial chemistry [54].

Applications:

The study lacks the biological data, but the compound 3-[1-(4-methylphenyl)-4,5-diphenyl 1H-imidazol-2-yl]-1H-indole has been evaluated by Nirwan et al. [53] for its antibacterial and anti-fungal activity, which was moderate when tested against *S. aureus* (ATCC 29213), *S. epidermidis* (ATCC 35984), *E. coli* (ATCC 25922), *P. aeruginosa* (ATCC 27853), and *C. albicans* (ATCC 10231).

## 3. Conclusions

The collective evidence indicates that indole–imidazole scaffolds represent a versatile class of heterocycles, distinguished by their broad therapeutic applications and innovative avenues of synthetic accessibility. Recent advances in multicomponent and metal-catalyzed reactions have not only expanded the chemical space but also improved reaction efficiency (reduced reaction times and improved yields) and functional diversity. Despite these advances, critical gaps persist, including limited scalability of synthetic methods, scarcity of in vivo evaluations, and lack of structural-activity relationship data, which collectively hinder the translation of many promising analogues into clinical applications. To overcome these challenges, future studies should focus on greener synthetic protocols, scalable synthetic routes, structural-activity relationship exploration, and robust pre-clinical evaluation of pharmacokinetics and toxicity. Furthermore, coupling computational drug design with high-throughput screening could significantly expedite the discovery and optimization of promising drug candidates. By bridging synthetic innovation with biological validation, indole–imidazole hybrids hold significant potential for the development of next-generation therapeutic agents against cancer, microbial infections, neurological disorders, and metabolic diseases. This review not only underscores their current achievements but also outlines future pathways, aiming to continue exploration of these highly promising molecular frameworks.

## Figures and Tables

**Table 1:** Growth inhibitory effects of ABI-III analogues on melanoma and prostate cancer cell lines.

Products	IC_50_ ± SEM (nM) (*n* = 4)					
	Melanoma and Prostate Cancer Cell Lines					
	A375	WM164	LNCaP	PC-3	Du 145	Average
6a	3.2 ± 1.2	5.3 ± 2.0	2.8 ± 0.6	3.7 ± 0.3	3.9 ± 1.0	3.8
6b	414.0 ± 35.4	166.2 ± 18.9	107.0 ± 17.5	105.0 ± 2.1	189.0 ± 13.5	196.2
6c	18.8 ± 3.6	24.2 ± 4.1	35.9 ± 3.3	40.7 ± 5.1	76.5 ± 4.1	39.8

**Table 2:** Antiproliferative activity of ABI-III analogues on paclitaxel-resistant cell lines.

Products	IC_50_ ± SEM (nM) (*n* = 4)		
	Paclitaxel-Resistant Cancer Cell Lines		
	PC-3	PC-3/TxR	Resistance Index
6a	3.7 ± 0.3	3.7 ± 0.8	1
6b	33.3 ± 2.1	27.6 ± 9.8	0.9
6c	20.8 ± 0.5	22.4 ± 5.1	1.1
Paclitaxel	0.4 ± 0.1	188.0 ± 22.0	437

**Table 3:** Antitumor activity of 2-phenyl indole derivatives on breast cancer cell lines.

Products	IC_50_ ± SEM (nM)	
	Breast Cancer Cell Lines	
	MCF-7	MDA-MB-231
9	23.50 ± 0.17	n.d.
10	28.40 ± 0.17	n.d.
Vincristine	2.00 ± 0.22	6.00 ± 0.14

n.d.: Not determined.

**Table 4:** Substituents on the starting materials (11) and (12), the indole–imidazole products (13), and their respective yields.

	Substituents on 11		Substituents on 12	Products (13)	Yield (%)
	Ar_1_	R_1_	R_2_		
a.	-C_6_H_5_	-H	-C_6_H_5_	3-(2,5-Diphenyl-1H-imidazol-4-yl) indolin-2-one	91
b.	-C_6_H_4_(4-CH_3_)	-H	-C_6_H_5_	3-(2-Phenyl-5-(p-tolyl)-1H-imidazol-4-yl) indolin-2-one	86
c.	-C_6_H_4_(4-OCH_3_)	-H	-C_6_H_5_	3-(5-(4-Methoxyphenyl)-2-phenyl-1H-imidazol-4-yl) indolin2-one	85
d.	-C_6_H_4_(4-F)	-H	-C_6_H_5_	3-(5-(4-Fluorophenyl)-2-phenyl-1H-imidazol-4-yl) indolin-2-one	85
e.	-C_6_H_4_(4-Cl)	-H	-C_6_H_5_	3-(5-(4-Chlorophenyl)-2-phenyl-1H-imidazol-4-yl) indolin-2-one	89
f.	-C_6_H_4_(4-Br)	-H	-C_6_H_5_	3-(5-(4-Bromophenyl)-2-phenyl-1H-imidazol-4-yl) indolin-2-one	87
g.	-THNp *(1,2,3,4-tetrahydronaphthalene)*	-H	-C_6_H_5_	3-(2-Phenyl-5-(5,6,7,8-tetrahydronaphthalen-2-yl)-1Himidazol-4-yl) indolin-2-one	84
h.	-C_6_H_5_	-CH_3_	-C_6_H_5_	3-(2,5-Diphenyl-1H-imidazol-4-yl)-5-methylindolin-2-one	87
i.	-C_6_H_4_(4-CH_3_)	-CH_3_	-C_6_H_5_	5-Methyl-3-(2-phenyl-5-(p-tolyl)-1H-imidazol-4-yl) indolin-2-one	81
j.	-C_6_H_4_(4-OCH_3_)	-CH_3_	-C_6_H_5_	3-(5-(4-Methoxyphenyl)-2-phenyl-1H-imidazol-4-yl)-5-methylindolin-2-one	90
k.	-C_6_H_3_(3,4-OCH_3_)_2_	-CH_3_	-C_6_H_5_	3-(5-(3,4-Dimethoxyphenyl)-2-phenyl-1H-imidazol-4-yl)-5-methylindolin-2-one	83
l.	-C_6_H_5_	-Cl	-C_6_H_5_	5-Chloro-3-(2,5-diphenyl-1H-imidazol-4-yl) indolin-2-one	93
m.	-C_6_H_4_(4-OCH_3_)	-Cl	-C_6_H_5_	5-Chloro-3-(5-(4-methoxyphenyl)-2-phenyl-1H-imidazol-4-yl) indolin-2-one	83
n.	-C_6_H_5_	-H	-2-(6-MeONp) *2-methoxy naphthalene*	3-(2-(6-Methoxynaphthalen-2-yl)-5-phenyl-1H-imidazol-4-yl) indolin-2-one	83
o.	-C_6_H_4_(4-CH_3_)	-H	-2-(6-MeONp) *2-methoxy naphthalene*	3-(2-(6-Methoxynaphthalen-2-yl)-5-(p-tolyl)-1H-imidazol-4-yl) indolin-2-one	79
p.	-C_6_H_4_(4-OCH_3_)	-H	-2-(6-MeONp) *2-methoxy naphthalene*	3-(2-(6-Methoxynaphthalen-2-yl)-5-(4-methoxyphenyl)-1Himidazol-4-yl) indolin-2-one	81
q.	-C_6_H_4_(4-F)	-H	-2-(6-MeONp) *2-methoxy naphthalene*	3-(5-(4-Fluorophenyl)-2-(6-methoxynaphthalen-2-yl)-1Himidazol-4-yl) indolin-2-one	91
r.	-C_6_H_4_(4-Cl)	-H	-2-(6-MeONp) *2-methoxy naphthalene*	3-(5-(4-Chlorophenyl)-2-(6-methoxynaphthalen-2-yl)-1Himidazol-4-yl) indolin-2-one	86
s.	-C_6_H_4_(4-OCH_3_)	-CH_3_	-2-(6-MeONp) *2-methoxy naphthalene*	3-(2-(6-Methoxynaphthalen-2-yl)-5-(4-methoxyphenyl)-1Himidazol-4-yl)-5-methylindolin-2-one	76
t.	-C_6_H_5_	-Cl	-2-(6-MeONp) *2-methoxy naphthalene*	5-Chloro-3-(2-(6-methoxynaphthalen-2-yl)-5-phenyl-1Himidazol-4-yl) indolin-2-one	84
u.	-C_6_H_5_	-H	-CH_3_	3-(2-Methyl-5-phenyl-1H-imidazol-4-yl) indolin-2-one	48

**Table 5:** Substituents on the starting materials (14) and (15), indole–imidazole products (16), and their respective yields.

	Substituents on 14			Substituents on 15	Products 16	Yield (%)
	R_1_	R_2_	R_3_	AR_1_		
a.	-H	-C_6_H_5_	-C_6_H_5_	-COOC_2_H_5_	Ethyl 1-Phenyl-5-(2-phenyl-1H-indol-1-yl)-1H-imidazole-4-carboxylate	62
b.	-H	-C_6_H_5_	-C_6_H_5_	-COOBu^t^	tert-Butyl 1-Phenyl-5-(2-phenyl-1H-indol-1-yl)-1H-imidazole-4-carboxylate	71
c.	-H	-C_6_H_5_	-C_6_H_5_	-(p-tolyl-sulphonyl)	1-Phenyl-5-(2-phenyl-1H-indol-1-yl)-4-(p-tolylsulfonyl)-1Himidazole	76
d.	-H	-C_6_H_4_(4-Cl)	-C_6_H_5_	-COOC_2_H_5_	Ethyl 5-[2-(4-Chlorophenyl)-1H-indol-1-yl]-1-phenyl-1H-imidazole-4-carboxylate	57
e.	-H	-C_6_H_4_(4-OCH_3_)	-C_6_H_5_	-COOC_2_H_5_	Ethyl 5-[2-(4-Methoxyphenyl)-1H-indol-1-yl]-1-phenyl-1Himidazole-4-carboxylate	66
f.	-H	-C_6_H_4_(4-CH_3_)	-C_6_H_5_	-COOC_2_H_5_	Ethyl 5-[2-(p-Tolyl)-1H-indol-1-yl]-1-phenyl-1H-imidazole-4-carboxylate	68
g.	-H	-C_3_H_5_ *(Cyclopropyl)*	-C_6_H_5_	-COOC_2_H_5_	Ethyl 5-(2-Cyclopropyl-1H-indol-1-yl)-1-phenyl-1H-imidazole-4-carboxylate	60
h.	-H	-C_6_H_5_	-C_6_H_4_(4-F)	-COOC_2_H_5_	Ethyl 1-(4-Fluorophenyl)-5-(2-phenyl-1H-indol-1-yl)-1H-imidazole-4-carboxylate	65
i.	-H	-C_6_H_5_	-C_6_H_4_(4-NO_2_)	-COOC_2_H_5_	Ethyl 1-(4-Nitrophenyl)-5-(2-phenyl-1H-indol-1-yl)-1H-imidazole-4-carboxylate	51
j.	-H	-C_6_H_5_	-C_6_H_4_(4-OCH_3_)	-COOC_2_H_5_	Ethyl 1-(4-Methoxyphenyl)-5-(2-phenyl-1H-indol-1-yl)-1H-imidazole-4-carboxylate	47
k.	-H	-C_6_H_5_	-C_6_H_4_(4-CH_3_)	-COOC_2_H_5_	Ethyl 5-(2-Phenyl-1H-indol-1-yl)-1-(p-tolyl)-1H-imidazole-4-carboxylate	64
l.	-H	-C_6_H_5_	-C_4_H_9_ *(n-butane)*	-COOC_2_H_5_	Ethyl 1-Butyl-5-(2-phenyl-1H-indol-1-yl)-1H-imidazole-4-carboxylate	60
m.	-CH_3_	-C_6_H_5_	-C_6_H_5_	-COOC_2_H_5_	Ethyl 5-(5-Methyl-2-phenyl-1H-indol-1-yl)-1-phenyl-1H-imidazole-4-carboxylate	60
n.	-Cl	-C_6_H_5_	-C_6_H_5_	-COOC_2_H_5_	Ethyl 5-(5-Chloro-2-phenyl-1H-indol-1-yl)-1-phenyl-1H-imidazole-4-carboxylate	61
o.	-H	-C_6_H_4_(4-Cl)	-C_6_H_5_	-(p-tolyl-sulphonyl)	1-Phenyl-5-[2-(4-chlorophenyl)-1H-indol-1-yl]-4-(p-tolylsulfonyl)-1H-imidazole	76
p.	-H	-C_6_H_4_(4-OCH_3_)	-C_6_H_5_	-(p-tolyl-sulphonyl)	1-Phenyl-5-[2-(4-methoxyphenyl)-1H-indol-1-yl]-4-(p-tolylsulfonyl)-1H-imidazole	78
q.	-H	-C_6_H_4_(4-CH_3_)	-C_6_H_5_	-(p-tolyl-sulphonyl)	1-Phenyl-5-[2-(p-tolyl)-1H-indol-1-yl]-4-(p-tolylsulfonyl)-1Himidazole	80
r.	-H	-C_6_H_5_	-C_6_H_4_(4-F)	-(p-tolyl-sulphonyl)	1-(4-Fluorophenyl)-5-(2-phenyl-1H-indol-1-yl)-4-(p-tolylsulfonyl)-1H-imidazole	85
s.	-H	-C_6_H_5_	-C_6_H_4_(4-OCH_3_)	-(p-tolyl-sulphonyl)	1-(4-Methoxyphenyl)-5-(2-phenyl-1H-indol-1-yl)-4-(p-tolylsulfonyl)-1H-imidazole	65
t.	-H	-C_6_H_5_	-C_6_H_4_(4-CH_3_)	-(p-tolyl-sulphonyl)	1-(p-Tolyl)-5-(2-phenyl-1H-indol-1-yl)-4-(p-tolylsulfonyl)-1Himidazole	67
u.	-H	-C_6_H_5_	-C_6_H_11_ *(Cyclohexyl)*	-(p-tolyl-sulphonyl)	1-Cyclohexyl-5-(2-phenyl-1H-indol-1-yl)-4-(p-tolylsulfonyl)-1H-imidazole	77
v.	-H	-C_6_H_5_	-C_4_H_9_ *(n-butane)*	-(p-tolyl-sulphonyl)	1-Butyl-5-(2-phenyl-1H-indol-1-yl)-4-(p-tolylsulfonyl)-1Himidazole	70
w.	-CH_3_	-C_6_H_5_	-C_6_H_5_	-(p-tolyl-sulphonyl)	5-(5-Methyl-2-phenyl-1H-indol-1-yl)-1-phenyl-4-(p-tolylsulfonyl)-1H-imidazole	81
x.	-Cl	-C_6_H_5_	-C_6_H_5_	-(p-tolyl-sulphonyl)	5-(5-Chloro-2-phenyl-1H-indol-1-yl)-1-phenyl-4-(p-tolylsulfonyl)-1H-imidazole	79
y.	-H	-C_6_H_4_(4-Cl)	-C_6_H_5_	-COOBu^t^	tert-Butyl 5-[2-(4-Chlorophenyl)-1H-indol-1-yl]-1-phenyl-1Himidazole-4-carboxylate	75

**Table 6:** Substituents on the starting materials (17), (18), and (21), indole–imidazole products (20) and (22), and their respective yields.

	Substituents on 17		Substituents on 18		Substituents on 21	Products		Yield (%)
	R_1_	R_2_	AR_1_	AR_2_	N			
a.	-H	-H	-H	-H	-	20a.	3-(1,4,5-Triphenyl-1H-imidazol-2-yl)-1H-indole	77
b.	-H	-H	-OCH_3_	-H	-	20b.	3-(1-(4-Methoxyphenyl)-4,5-diphenyl-1H-imidazol-2-yl)-1H-indole	78
c.	-H	-H	-OC_2_H_5_	-H	-	20c.	3-(1-(4-Ethoxyphenyl)-4,5-diphenyl-1H-imidazol-2-yl)-1H-indole	81
d.	-H	-H	-OCH_3_	-OCH_3_	-	20d.	3-(1-(3,4-Dimethoxyphenyl)-4,5-diphenyl-1H-imidazol2-yl)-1H-indole	78
e.	-H	-Br	-H	-H	-	20e.	5-Bromo-3-(1,4,5-triphenyl-1H-imidazol-2-yl)-1H-indole	57
f.	-Br	-Br	-OCH_3_	-H	-	20f.	2,5-Dibromo-3-(1-(4-methoxyphenyl)-4,5-diphenyl1H-imidazol-2-yl)-1H-indole	66
g.	-H	-Br	-OC_2_H_5_	-H	-	20g.	5-Bromo-3-(1-(4-ethoxyphenyl)-4,5-diphenyl-1H-imidazol2-yl)-1H-indole	66
h.	-CH_3_	-H	-H	-	-	20h.	2-Methyl-3-(1,4,5-triphenyl-1H-imidazol-2-yl)-1H-indole	80
i.	-CH_3_	-H	-OC_2_H_5_	-	-	20i.	3-(1-(4-Ethoxyphenyl)-4,5-diphenyl-1H-imidazol-2-yl)-2-methyl-1H-indole	84
j.	-	-	-	-	1	22a.	1,4-Bis(3-(1,4,5-triphenyl-1H-imidazol-2-yl)-1H-indol1-yl) butane	87
k.	-	-	-	-	2	22b.	1,5-Bis(3-(1,4,5-triphenyl-1H-imidazol-2-yl)-1H-indol1-yl) pentane	93
l.	-	-	-	-	3	22c.	1,6-Bis(3-(1,4,5-triphenyl-1H-imidazol-2-yl)-1H-indol1-yl) hexane	91

**Table 7:** Anti-microbial activity of mono-indolyl imidazoles and bis-indolyl imidazoles.

Products	Anti-Microbial Activity (Zone of Inhibition in mm)			
	*P. aeruginosa*	*S. enteritis*	*B. subtilis*	*M. Luteus*
20a	-	-	10	25
20b	-	13	11	21
20c	-	-	-	14
20d	-	-	12	-
20e	-	-	13	17
20f	-	-	9	11
20g	-	-	11	15
20h	-	-	11	14
20i	-	-	16	18
22a	-	11	10	13
22b	-	-	-	-
22c	-	-	9	-
Erythromycin	10	8	12	10
Tetracycline	18	7	14	16

**Table 8:** Substituents on the starting materials (23) and (24), indole–imidazole products (27), and their respective yields.

	Substituents on 23		Substituent on 24	Products 27	Yield (%)
	R_1_	R_2_	AR_1_		
a.	-H	-Cl	-CH_3_	2-(p-Chlorophenyl)-3-(4,5-diphenyl-1-(p-tolyl)-1H-imidazol-2-yl)-1Hindole	57
b.	-H	-Cl	-Br	3-(1-(p-Bromophenyl)-4,5-diphenyl-1H-imidazol-2-yl)-2-(p-chlorophenyl)-1H-indole	68
c.	-Br	-H	-F	5-Bromo-3-(1-(p-fluorophenyl)-4,5-diphenyl-1′H-imidazol-2-yl)-2-phenyl-1H-indole	83
d.	-H	-Br	-CH_3_	2-(p-Bromophenyl)-3-(4,5-diphenyl-1-(p-tolyl)-1H-imidazol-2-yl)-1Hindole	52
e.	-H	-Br	-CL	2-(p-Bromophenyl)-3-(1-(p-chlorophenyl)-4,5-diphenyl-1H-imidazol2-yl)-1H-indole	86
f.	-H	-CH_3_	-H	2-(p-Tolyl)-3-(1,4,5-triphenyl-1H-imidazol-2-yl)-1H-indole	54
g.	-H	-CH_3_	-CH_3_	3-(4,5-Diphenyl-1-(p-tolyl)-1H-imidazol-2-yl)-2-(p-tolyl)-1H-indole	68
h.	-H	-CH_3_	-Br	3-(1-(p-Bromophenyl)-4, 5-diphenyl-1H-imidazol-2-yl)-2-(p-tolyl)-1Hindole	48
i.	-H	-CH_3_	-F	3-(1-(p-Fluorophenyl)-4,5-diphenyl-1H-imidazol-2-yl)-2-(p-tolyl)-1Hindole	54
j.	-H	-CH_3_	-OCH_3_	3-(1-(p-Methoxyphenyl)-4,5-diphenyl-1H-imidazol-2-yl)-2-(p-tolyl)-1H-indole	46

**Table 9:** Anti-urease and antioxidant activity of tetra aryl imidazole and indole derivatives.

Products	Anti-Urease Activity		Antioxidant Activity	
	Inhibition (%)	IC_50_ (µM)	Inhibition (%)	IC_50_ (µM)
27a	76.44 ± 0.8	47.53 ± 0.12	62.58 ± 0.7	175.26 ± 1.24
27b	76.54 ± 0.8	47.64 ± 0.15	71.74 ± 0.2	146.27 ± 1.09
27c	92.58 ± 0.3	11.35 ± 0.07	71.87 ± 0.5	181.26 ± 1.1
27d	87.44 ± 0.8	9.95 ± 0.05	90.39 ± 0.5	148.26 ± 1.2
27e	89.44 ± 0.3	20.25 ± 0.03	20.97 ± 0.5	-
27f	85.77 ± 0.6	4.85 ± 0.07	67.61 ± 0.3	162.27 ± 1.2
27g	86.73 ± 0.7	0.16 ± 0.04	44.21 ± 0.7	-
27h	87.38 ± 0.46	1.45 ± 0.05	7.11 ± 0.2	-
27i	89.73 ± 0.9	4.31 ± 0.02	18.91 ± 0.6	-
27j	87.63 ± 0.9	0.12 ± 0.02	23.03 ± 0.5	-
Thiourea	88.7 ± 0.8	21.25 ± 0.15	-	-
Quercetin	-		93.21 ± 0.9	16.96 ± 0.1

**Table 10:** Substituents on the starting materials (28) and (29); indole–imidazole products (30), and their respective yields.

	Substituents on 28		Substituents on 29	Products 30	Yields (%)
	R_1_	R_2_	AR_1_		
a.	-H	-H	-H	2-Imidazol-1-yl-1H-indole	90
b.	-H	-CH_3_	-H	2-Imidazol-1-yl-1-methyl-1H-indole	64
c.	-H	-CH_3_	-CH_3_	1-Methyl-2-(2-methyl-imidazol-1-yl)-1H-indole	61

**Table 11:** Substituents on the starting material (32), the indole–imidazole products (33) and (35), and their respective yields.

	Substituents on 32			Product 33	Yields (%)
	R_1_	R_2_	R_3_		
a.	-H	-H	-CH_3_	3-((2-methyl-1H-imidazol-1-yl) methyl)-1H-indole	85
b.	-H	-H	-C_2_H_5_	3-((2-ethyl-1H-imidazol-1-yl) methyl)-1H-indole	89
c.	-H	-H	-CH(CH_3_)_2_	3-((2-isopropyl-1H-imidazol-1-yl) methyl)-1H-indole	88
d.	-H	-CH_3_	-C_2_H_5_	3-((2-ethyl-4-methyl-1H-imidazol-1-yl) methyl)-1H-indole	69
e.	-Cl	-Cl	-H	3-((4,5-dichloro-1H-imidazol-1-yl) methyl)-1H-indole	50
f.	-H	-H	-C_6_H_5_	3-((2-phenyl-2,5-dihydro-1H-imidazol-1-yl) methyl)-1H-indole	56
35.				1-((1H-indol-3-yl)methyl)-1H-benzo[d]imidazol	95

**Table 12:** Antibacterial activity of indolyl methane–imidazole derivatives.

Products	Antibacterial Activity (Zone of Inhibition in mm)			
	*M. Luteus*	*B. subtilis*	*E. coli*	*P. fluorescens*
33a	3	1	1	16
33b	8.8	4.3	3.8	3
33c	3	1.8	3	0
33d	6	2.4	3.5	2.3
33e	5	8.7	4.3	0
33f	8.6	3.7	5	0
35	4.3	2.7	3	0

**Table 13:** Anti-fungal activity of indolyl methane–imidazole derivatives.

Products	Concentration of Products	Fungicidal Effect Inhibition (%)			
	**(%)**	*C. puteana*	*G. trabeum*	*P. placenta*	*C. versicolor*
33a	0.1	100	100	100	100
	0.01	48	44	48	35
	0.001	0	19	0	1
	0.0001	0	15	0	0
33b	0.1	100	100	100	100
	0.01	37	30	32	43
	0.001	15	11	18	13
	0.0001	13	8	16	6
33c	0.1	100	100	100	100
	0.01	26	17	21	43
	0.001	14	16	6	12
	0.0001	13	12	1	13
33d	0.1	100	100	100	100
	0.01	49	27	41	44
	0.001	37	9	0	19
	0.0001	40	13	4	19
33e	0.1	100	100	100	100
	0.01	74	91	78	96
	0.001	46	51	35	23
	0.0001	0	28	0	0
33f	0.1	100	100	100	100
	0.01	48	53	45	41
	0.001	0	18	17	13
	0.0001	0	13	0	7
35	0.1	100	100	100	100
	0.01	40	48	37	44
	0.001	0	28	23	6
	0.0001	0	17	0	0

**Table 14:** Substituents on the starting materials (36) and (38), the indole–imidazole products (39), and their respective yields.

	Substituents on 36	Substituents on 38	Products 39	Yield (%)
	R	AR_1_		
a.	-H	-H	3-(4,5-diphenyl-1H-imidazol-2-yl)-1H-indole	97
b.	-Br	-H	5-bromo-3-(4,5-diphenyl-1H-imidazol-2-yl)-1H-indole	92
c.	-Cl	-H	5-Chloro-3-(4,5-diphenyl-1H-imidazol-2-yl)-1H-indole	89
d.	-I	-H	5-Iodo-3-(4,5-diphenyl-1H-imidazol-2-yl)-1H-indole	90
e.	-H	-C_6_H_5_	3-(1,4,5-triphenyl-1H-imidazol-2-yl)-1H-indole	94
f.	-H	-C_6_H_4_(4-CH_3_)	3-(4,5-diphenyl-1-(p-tolyl)-1H-imidazol-2-yl)-1H-indole	93
g.	-H	-C_6_H_4_(4-OCH_3_)	3-(1-(4-methoxyphenyl)-4,5-diphenyl-1H-imidazol-2-yl)-1H-indole	96
h.	-H	-C_6_H_4_(4-OC_2_H_5_)	3-(1-(4-ethoxyphenyl)-4,5-diphenyl-1H-imidazol-2-yl)-1H-indole	97

**Table 15:** Anti-microbial activity of indolyl-imidazole derivatives.

Products	Anti-Microbial Activity (Zone of Inhibition in Mm)				
	*S. aureus* ATCC 29213	*S. epidermidis* ATCC 35984	*E. coli* ATCC 25922	*P. aeruginosa* ATCC 27853	*C. albicans* ATCC 10231
39a	13.32 ± 12	07.62 ± 51	10.66 ± 21	18.96 ± 21	08.14 ± 11
39b	16.12 ± 22	14.33 ± 05	14.06 ± 62	17.66 ± 37	09.59 ± 03
39c	20.68 ± 31	18.68 ± 30	18.54 ± 32	13.54 ± 08	10.24 ± 07
39d	15.68 ± 68	11.82 ± 02	08.98 ± 11	10.44 ± 32	11.55 ± 41
39e	08.20 ± 38	09.38 ± 09	12.22 ± 06	09.24 ± 64	09.74 ± 03
39f	07.57 ± 41	05.62 ± 16	05.52 ± 45	08.49 ± 09	06.30 ± 03
39g	08.98 ± 82	06.38 ± 15	08.44 ± 27	12.87 ± 61	08.37 ± 83
39h	05.64 ± 76	08.58 ± 55	07.57 ± 87	09.68 ± 69	06.82 ± 05
Ampicillin	15.03 ± 38	15.00 ± 53	07.53 ± 08	12.09 ± 09	-
Amikacin	19.03 ± 6	17.69 ± 08	18.13 ± 64	18.82 ± 62	-
Fluconazole	-	-	-	-	21.23 ± 06

## Data Availability

No new data were created or analyzed in this study. Data sharing is not applicable.
